# Impact of Waste Cooking Oils Addition on Thermophilic Dry Co-Digestion of Wheat Straw and Horse Manure for Renewable Energy Production in Two Stages

**DOI:** 10.3390/life14030312

**Published:** 2024-02-27

**Authors:** Venelin Hubenov, Iva Varbacheva, Lyudmila Kabaivanova

**Affiliations:** The Stephan Angeloff Institute of Microbiology, Bulgarian Academy of Sciences, 1040 Sofia, Bulgaria; varbachevaiva@gmail.com

**Keywords:** agricultural wastes, thermophilic co-digestion, renewable energy, waste cooking oils

## Abstract

Anaerobic co-digestion of waste wheat straw and horse manure in two steps was revealed as a promising option for renewable energy production in the form of hydrogen and methane. Addition of waste cooking oils, disposal of which could cause damage to health or the environment, as a third substrate for digestion, is suggested as an approach not only to help handle the increasing volume of food waste worldwide but also to improve process performance. In the present study, waste cooking oil, in a concentration of 5%, appeared to be a positive modulator of anaerobic digestion with the production of hydrogen and did not lead to inhibition of the hydrolysis phase. The overall efficiency of the two-stage anaerobic digestion of the mixture, which contains mainly lignocellulose waste, is positively dependent on thermochemical pretreatment with the alkali reagent (Ca(OH)_2_), but elevated temperature (55 °C) and cooking oil addition revealed the opportunity to omit the pre-treatment step. Nevertheless, the overall energy production was lower due to the methane production step. However, the addition of waste cooking oils to the process in which lig-nocellulose is not pretreated (V3) led to an increase in the methane production and energy yield compared to V1. The anaerobic digestion of lignocellulosic waste is a complex process and comprises successive degradation pathways and syntrophic microbial associations’ activities, so the division in two reactors ensured suitable conditions for the microorganisms residing in each of them. In this study, along with the production of hydrogen and methane and the separation of the hydrolysis and methanogenesis stages, utilization of agriculture- and kitchen-generated wastes was realized in the context of waste-to-energy sustainable production methods.

## 1. Introduction

Anaerobic digestion is a process in which energy is generated in the form of biogas—namely methane and hydrogen. This method allows the utilization of multiple products, such as nutrients, enzymes, carboxyl acids, bioplastic, bio-oil, waxes, and so on [1]. The development of technologies for utilizing new energy sources has led to the introduction of some of these into practice. The technology of anaerobic biodegradation of organic waste will increasingly find its place, especially near locations where large volumes of biodegradable waste have been generated [2]. It should be properly integrated into production systems where energy can be recovered from organics [3]. The growing problems of global warming, depletion of the ozone layer, and formation of acid rains could have a solution if the use of fossil fuels as sources of energy is replaced by energy obtained through anaerobic digestion [4]. Hydrogen is considered a potential form of energy due to its reduced greenhouse gas emissions. Production of hydrogen by biological means is an environmentally friendly method in which the sustainable form of energy is obtained from organic carbon materials [5]. Due to the need for alternative sources of energy, the biological production of methane and hydrogen, as renewable energy sources, from biomass is now highly regarded [6]. Biotechnological processes for obtaining biofuels can be assigned to the so-called white biotechnologies, which are focused on the obtaining and processing of chemicals, materials, and energy through the action of living cells (microbial communities) [7].

Lignocellulosic biomass from agriculture and waste from the food industry (animal fat or cooking waste, such as waste cooking oils) are the main raw material sources for second-generation biofuels. The need for diversified low-cost energy sources will play an important role in the production of bioenergy and green chemicals [8]. Although very common and largely suitable for input as raw materials in the processes of anaerobic degradation, some agricultural wastes are difficult to use for effective biodegradation alone. Several studies have shown that methane production from lignocellulosic wastes and process performance of anaerobic digestion can be enhanced by co-digestion with various substrates [9]. Therefore, establishing the appropriate ratios for their mixing represents an interesting object of study, taking into account the need for extensive pretreatment of these materials due to their complex structure [10]. Agricultural waste can be united in the concept of “biomass”. Lignocellulosic biomass is the most common and constitutes about 70% of the total plant biomass. The main polymer molecules making up the lignocellulosic biomass are cellulose, hemicellulose, and lignin, which interact with each other and form a very tough structure [11]. Cooking oil is one the most essential components in food preparation around the world [12]. Its accumulation could cause some serious environmental problems, but sustainable production methods for new-generation fuels and hydrogen are now being intensively studied in view of the potential reduction in greenhouse gas emissions.

For the most part, the research related to anaerobic biodegradation of organic waste and methane and/or hydrogen production refers to processes that take place at relatively low concentrations of organic matter (up to about 10% dry weight). These processes are particularly suitable for waste streams with a high water content (e.g., activated sludge) [13]. Other raw materials, such as wheat straw, are quite low in moisture, and their use in anaerobic digestion processes would require additional consumption of fresh water. For this reason, solid-phase fermentation is exclusively applicable to this type of raw material [14]. In practical terms, anaerobic digestion systems are exceptional but present concerns with regard to the size and configuration of bioreactors. For a more complete utilization of the raw materials, reduction of the retention time, and increase in the overall energy balance, cascade systems can be proposed in order to intensify the processes [15]. A natural development of these cascade systems is the two-stage systems for simultaneous production of hydrogen and methane, in which the effluent from the first stage of the degradation and production of hydrogen is usually used as a feedstock in the second stage, where the target product is methane. These systems offer physical separation of the two processes and provide the necessary conditions for each of them. In addition, a good possibility is the application of different temperature regimes in each of the reactors, as well as the possibility of realizing the so-called temperature-phased processes [16]. With them, anaerobic digestion is realized under different temperature conditions in different reactors, usually connected in series by the effluent streams. The aim of these systems is to combine the advantages of both processes, improving the stability of processes and gas yields. Finally, yet importantly from the point of view of applying the principles of circular economy and the possibilities for the application of spent nutrient media digestate in agriculture, these systems with temperature phasing would help suppress pathogenic microorganisms.

The aim of present study was to investigate the possibilities of conveying two-stage thermophilic dry fermentation of a mixture of wheat straw and horse manure and to determine the influence of pre-treatment of wheat straw as well as addition of waste cooking oils, by estimating the potential of sequential production of the energy carriers hydrogen and methane.

## 2. Materials and Methods

### 2.1. Experimental Setup

The experimental setup was composed of four reaction vessels placed in a water thermostat with automatic temperature control. Each of the reaction vessels has an adjacent gas holder, representing a graduated glass cylinder filled with water and placed with the opening downwards in a plastic container. About 1/3 of its volume is also filled with water. The reaction vessels have a total volume of 1.2 dm^3^ and work volume of 0.5 dm^3^. The vessels are hermetically closed with the help of rubber stoppers (Saint Gobain Life Sciences Pre-Drilled Conical Rubber Stoppers). In two holes, hoses are placed in the stoppers to remove the product obtained during the fermentation process. The gas goes to the gas holder, and there is a connection to a gas analyzer to determine its composition. During the processes for anaerobic biodegradation (for obtaining hydrogen, and subsequently, methane) the temperature was maintained at 55 ± 1 °C. The effect of addition of waste cooking oil to the basic feedstocks was evaluated by the introduction of 5% of it to one of the variants tested with pretreated straw (V4) and to one of the variants where straw was used without pretreatment (V3) (Table 1)

### 2.2. Raw Materials and Mixtures Thereof

The following was used as a starting raw material: wheat straw, mechanically treated to reduce particle size to 3–5 mm using a hammer mill. In two of the variants, it was treated thermochemically in advance, and in the other two, it was applied without pretreatment. Pretreatment consisted of mixing the required amount of straw with a solution of Ca(OH)_2_ in a ratio of 4 g Ca(OH)_2_/100 g of wheat straw (in terms of dry matter), followed by heat treatment at 55 °C for 24 h immediately before starting of periodic experiments. Horse manure was also mechanically pretreated by grinding with a knife mill to a particle size below 5 mm. Waste cooking oil was used without processing, in the form in which it was provided by catering establishments. For this raw material, 5% (wt.) was added to two of the variants. Wheat straw and horse manure were provided by a horse farm near the city of Burgas, Bulgaria.

The two main raw materials were used in the form of a mixture in a percentage ratio wheat straw:horse manure = 55:45. In two of the variants, the wheat straw was mechanically and thermochemically pretreated.

The effect of addition of waste cooking oils to the basic feedstocks was evaluated by the addition of 5% cooking oils to one of the options with pre-treated straw and to one of the options in which straw was used without pretreatment (Table 1).

### 2.3. Inoculum

In both processes, for inoculation, inoculum from a working methanogenic process was used. For the hydrogen-generating process, it was subjected to pre-treatment in order to remove methanogens and with no pretreatment for the methane-generating second step.

Treatment of the starter inoculum for the hydrogen production was performed as follows: Aliquots of 100 mL of the liquid were taken after straining to remove the solid particles through a 1 mm sieve. Microbial biomass in the aliquots was collected by centrifugation at 4500 rpm for 10 min. The resulting pellet was washed twice with sterile saline and resuspended in a new portion of saline to a final volume of each aliquot of 50 mL. This was followed by heat treatment in a water thermostat at 80 °C for 30 min [17]. In this way, the treated inoculum was adjusted to pH 5.5 with 1 M HCl and was ready for inoculation of the reaction vessels.

### 2.4. Analytical Methods

The content of the components included in the composition of the emitted gas was measured using the following devices: (1) gas analyzer model “Gasboard 3100P” (Cubic Sensor and Instrument Co., Ltd., Wuhan, China) to measure H_2_ and CO_2_ contents in volume percentages using infrared sensors; and (2) gas analyzer model X-am 7000 of the Dräger company (Singapore), equipped with infrared detectors for CH_4_ and CO_2_ and a catalytic sensor for H_2_S.

### 2.5. Cellulose

Quantification of the cellulose present in the samples was performed according to the spectrophotometric method, based on the ability of reducing sugars to reduce anthrone in a strongly acidic medium. Samples were processed as suggested by the Updegraff methodology [18]. The quantity of cellulose was determined by a standard curve made with microcrystalline cellulose (Avicel PH101, Sigma-Aldrich, St. Louis, MI, USA).

### 2.6. Total Solids and Volatile Solids

The total solids (TS) content was determined by weight, according to [19].

Determination of the volatile solids (VS) in the raw materials and in the samples was determined according to [20].

### 2.7. Volatile Fatty Acids Measurement

The composition and concentrations of the volatile fatty acids (VFA) obtained during the processes (acetic, oleic, iso-oleic, propionic, valerian, iso-valerian, and caproic) were determined with a Thermo Scientific (Waltham, MA, USA) gas chromatograph, model Focus GC, equipped with Split/Splitless injector, flame ionization detector (FID), and TG-WAXMS A column with a length of 30 m, diameter of 0.25 mm, and film thickness of 0.25 µm.

### 2.8. pH

The pH was determined using a microprocessor pH meter model “pH210” (HANNA Instruments, Woonsocket, RI, USA).

### 2.9. Calculation of the Potential for Obtaining of Energy

The resulting primary energy after the sequential production of hydrogen and methane using the proposed mixture of horse manure and wheat straw was calculated using the following Equation (1):

*E_e_* = *Y* × *LHV* × *η_e_* × (*m_p_*/*m_u_*) (1)

where *Y* is the methane yield in m^3^·t^−1^, the *LHV* (lower heating value) is taken to be 9.94 kWh·Nm^−3^ (for methane) and 2.99 kWh·Nm^−3^ (for hydrogen), *η_e_* is the electrical efficiency of the cogenerator rated at 40%, and *m_u_* and *m_p_* are the masses of the sample before and after pretreatment [21].

### 2.10. Statistical Analysis

All analyses and tests were performed in triplicate. The statistical analyses were carried out using Microsoft Excel 2016. A one-way analysis of variance (ANOVA) test was used to determine levels of confidence among various results. All the results were expressed as means ± standard deviations, and the *p*-value was considered to be significant at *p* < 0.05.

## 3. Results and Discussion

The effect of pretreatment and addition of waste cooking oil to a mixture of wheat straw and horse manure on the hydrogen and methane production in two steps was investigated. After cessation of gas separation from the reaction vessels, in which the anaerobic biodegradation for the production of hydrogen had taken place, the rubber plugs were removed, and the mixture, already partially processed, was well homogenized. Samples of 50 mL each were taken for analysis of pH, residual total solids, volatile solids, cellulose, and volatile fatty acids accumulated during the first step. Then, pH adjustment was carried out to 7.5 (with 2 M NaOH), followed by addition of inoculum (50 mL) from a working methanogenic process, ensuring anaerobic conditions. For the purpose of waste materials utilization, mixed microbial communities are a good solution, which, however, require preliminary treatment due to the co-evolution of hydrogen-producing and hydrogen-consuming bacteria. The methods applied for pretreatment of the mixed microbial communities include thermal, chemical, microwave treatment, or aeration. Because of the facilitated application and its effectiveness, methods involving thermal treatment are preferred for starting anaerobic biodegradation processes for the production of hydrogen [22].

For implementation of the anaerobic biodegradation processes for the production of hydrogen, as well as of methane, a culture with the same source was used—a laboratory bioreactor operating under as close as possible conditions to the studied ones and with a raw material of alkaline-pretreated wheat straw. The only difference between the cultures applied to obtain hydrogen is in the pretreatment, aiming at complete suppression of the activity of methanogenic microorganisms.

Agricultural waste, as the potentially most widespread and suitable substrate for anaerobic digestion processes for energy production, was used. The main indicators of the two raw materials used are shown in Table 2.

The dry substance of wheat straw exceeds that of horse manure by more than double and is characterized by a higher cellulose content. Horse manure has a moisture content of approximately 63%, but at the same time, wheat straw has a moisture content of about 7%. The total amount of used raw materials gives a dry substance content of about 29% in the reaction mixture, which according to some authors approaches extremely dry conditions (22–40%) [23]. Lignocellulosic substrates are complex, as the typical composition of lignocellulosic materials comprises 10–25% lignin, 40–50% cellulose, and 5–30% hemicellulose [24]. Based on these indicators, calculations were made to observe the necessary ratio between the raw materials in the final mixture. The measurement data were similar to those reported by other authors [25].

The use of wheat straw and horse manure, which accumulate and are found in large quantities, could be improved by preliminarily wheat straw treatment. The introduction of additional substances, such as animal manure in the biodegradation system, would help stabilize the process and lead to more complete conversion of the introduced dry matter. Because of the simultaneous presence of lignin and of crystalline areas in cellulose molecules, water cannot easily penetrate the lignocellulosic fibers. In addition to mechanical rigidness, water-insoluble lignocelluloses also have resistance to enzymatic effects. Lignin preserves and strengthens the fibers, inhibiting the action of the enzymes, but the crystalline areas of the cellulose also decrease the surface area accessible to enzymes. However, the presence of lignin in the feedstock could affect energy production, and efficient pretreatment becomes mandatory for maximum lignin removal [26]. Therefore, preliminary thermochemical treatment of lignocellulosic raw material (wheat straw) was carried out with Ca(OH)_2_ and elevated temperature (55 °C) after being mechanically treated for particle size reduction to a size of 3–5 mm using a hammer mill. Pretreatment technologies can increase process efficiency [27].

Waste cooking oils can be considered as a potential waste that can be utilized as an energy source and raw material for chemical or biological purposes in fermentation processes [28]. Due to their composition of triglycerides, untreated waste cooking oils can be used as feedstock for microbial growth as a carbon source [29]. A sustainable biotechnological approach to energy production is the newest means for efficient waste management and utilization of waste products [30].

The production of hydrogen and methane was realized in two successive stages, which had its advantages over the single anaerobic digestion process [31]. For the implementation of the anaerobic digestion process with hydrogen production, initial conditions were set at pH 5.5.

**Figure 1:**
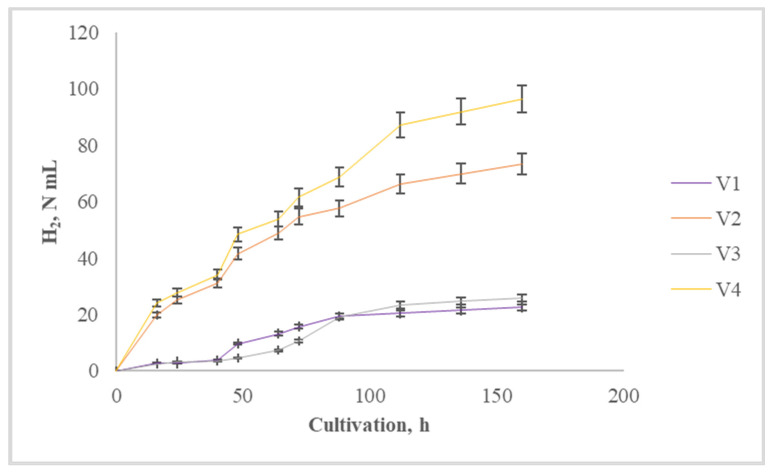
Cumulative hydrogen yield during digestion at 55 °C.

For the variants in which the wheat straw was subjected to thermochemical pretreatment, a hydrogen yield of 70–100 N mL at the 150th hour after the start of the process was achieved (Figure 1). Highest was the yield where, together with straw pretreatment, 5% waste cooking oil was included in the substrate mixture (V4). Although significantly lower, maximum yields of hydrogen in the other two variants were achieved at the same time. Due to pretreatment of the wheat straw, the polymer molecules were more susceptible to attack by microorganisms. Different pretreatment techniques have been applied to lignocellulosic substrates, and the effect of hydrothermal pretreatment on the digestibility of wheat straw was investigated under different temperatures (160–180 °C) and retention times (15–45 min); a significant increase in biomethane potential was observed in all cases [32].

Microorganisms producing hydrogen also have other end products such as acetic, lactic, butyric, and propionic acid. Their presence also leads to a decrease in pH and hence inhibition of the activities of the microorganisms themselves. Acetic and butyric acid increase the yield of hydrogen. As for the acetate or acetone, the obtaining of these end products gives the highest theoretical yield of hydrogen: 4 moles per mole of glucose. Formiate and butyrate yield 2 moles of hydrogen per mole of glucose. Alcohols, as an end product, in turn contain additional hydrogen atoms that do not transform to hydrogen, and as a result, the yield becomes lower. That is why the production of ethanol, butanol, and lactic acid do not lead to hydrogen production. Therefore, the accumulation of volatile fatty acids and the avoidance of obtaining reduced products, such as lactate and alcohols, are of utmost importance [1].

**Figure 2:**
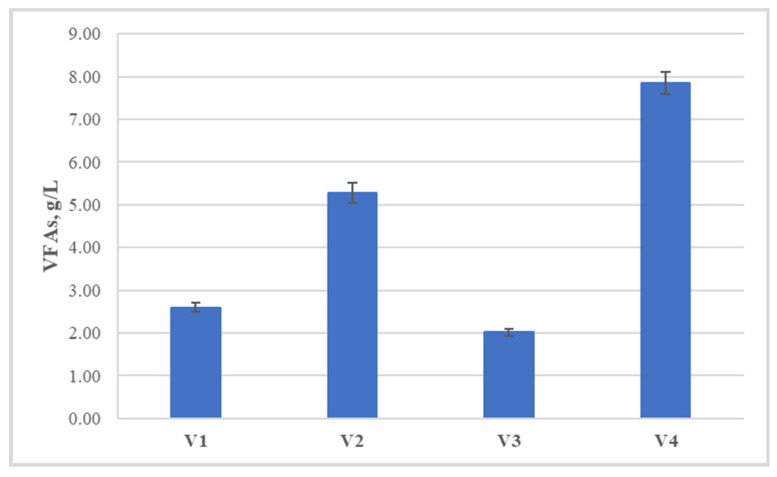
Total concentration of volatile fatty acids at the end of the hydrogen generation process.

The data obtained from the gas-chromatographic determination of the concentration of volatile fatty acids (Figure 2) also support the assumption of facilitated hydrolysis and acidogenesis, as in variant V2, their total concentration is about 5 g/L, and in V4, a little above 7.5 g/L. The main component of the accumulated volatile fatty acids is acetate, which is one of the end products, showing stable running of the process and directing it towards hydrogen production. In the other two variants, the concentrations of acetate and butyrate are quite close, and the total amounts are 2.60 g/L and 2.01 g/L, respectively.

The target end product, hydrogen, can sometimes have a negative impact on the process with an increase in its partial pressure in the nutrient medium. One possible reason for this phenomenon is the shift of the metabolic pathway towards obtaining other highly reduced compounds such as acetone, butane, ethanol, and others. The increased partial pressure of hydrogen strongly limits the uptake of the substrate, since the reduced ferredoxin and the hydrogen-transferring co-enzymes are reoxidized by the bacteria due to hydrogen production. In order to increase the yield of biohydrogen, it is necessary to ensure continuous evacuation of the gas after its separation. One of the advantages of the realization of anaerobic digestion at higher temperatures (suitable for thermophilic microorganisms) is the smaller influence of this factor on the system. Other methods of preventing the increase in the partial pressure of hydrogen are acceleration of stirring or the addition of an inert gas to the system [33].

**Figure 3:**
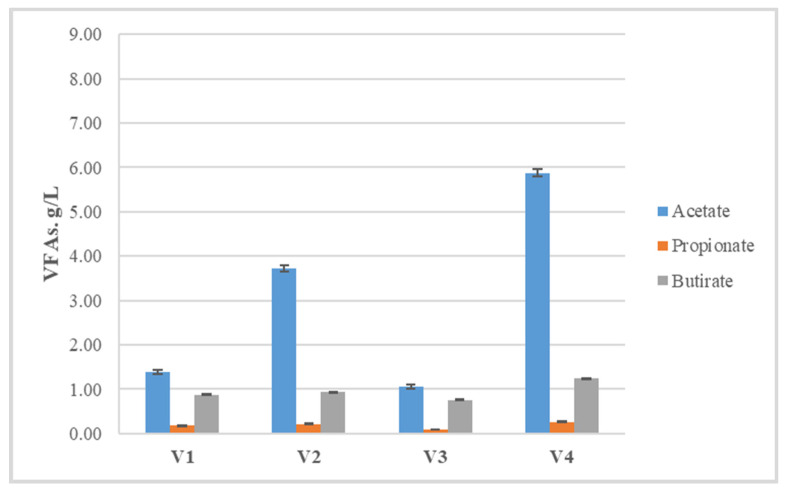
Concentrations of volatile fatty acids with the highest content at the end of hydrogen generation process.

In all variants, the concentration of propionic acid was the lowest (Figure 3), which results in a favorable ratio between propionate and acetate, below 1.4 (Table 3). This indicator is important for the next methane-generating step, as the spent cultivation medium from the hydrogen generation process becomes the starting medium for the methane generation process, and starting conditions that are suitable for the development of methanogenic microorganisms have to be ensured.

In the variants in which the wheat straw is not pretreated, the butyrate isoforms and valerate, together with valeric acid itself, are not detected (Table 3).

**Figure 4:**
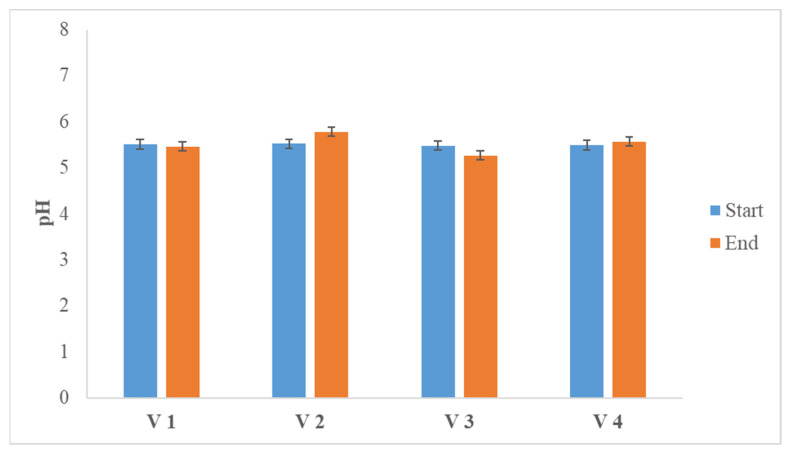
pH values at the start and at the end of the hydrogen generation process.

Despite the acids accumulated during the process, the pH of the proposed mixture of wheat straw and horse manure deviates only slightly from the initial set value of 5.5, and these deviations are within the limits in which the process is not negatively affected, due to strong acidification of the medium (Figure 4).

Volatile fatty acids, and in particular acetic acid, apart from a final metabolic product in the processes of anaerobic digestion for the production of hydrogen, are also a substrate for the methanogenic microorganisms in the next methane production process, and it can be expected that the process will start more intensively in variants V2 and V4.

After the completion of gas separation in the process of obtaining hydrogen, the pH of each variant was adjusted to a value close to 7.5, inoculum (10% vol) from a working methanogenic process was added, and the anaerobic conditions were restored for the process of methane production.

**Figure 5:**
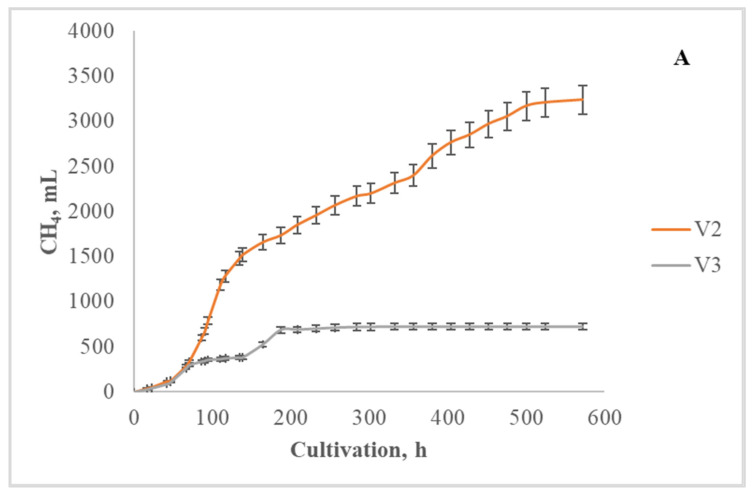

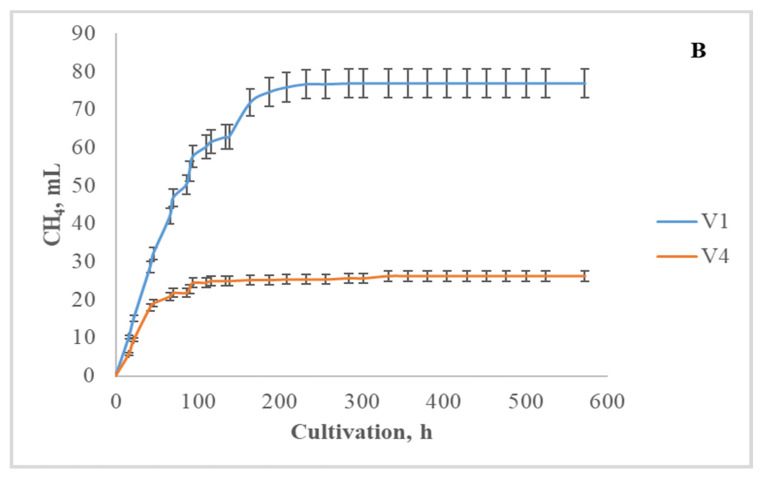
Cumulative methane yield during methane production process at 55 °C of variants V2 and V3 (**A**) and V1 and V4 (**B**).

Variant 2 (V2) showed the best results, in view of the high yield of methane and effective reduction of volatile fatty acids concentration. The total methane yield was above 3000 NmL in the variant with straw pretreatment, but with the addition of waste cooking oil, the yield was lower (Figure 5A).

In the other two variants, the intensive gas production was up to 200 h from the start of the process, after which gas evolution almost stopped (Figure 5B). In other studies, the addition of a certain amount of waste cooking oil usually has a positive effect on methane yields, with an increase of the yield reported in some cases [34], compared to the variants without the addition of waste cooking oil. The differences with the present study may be due to the different temperature at which the process is run or the fact that in the cited study the process is carried out in one stage. The presence of hydrogen sulfide is greater in the variants with the lowest methane yield.

**Figure 6:**
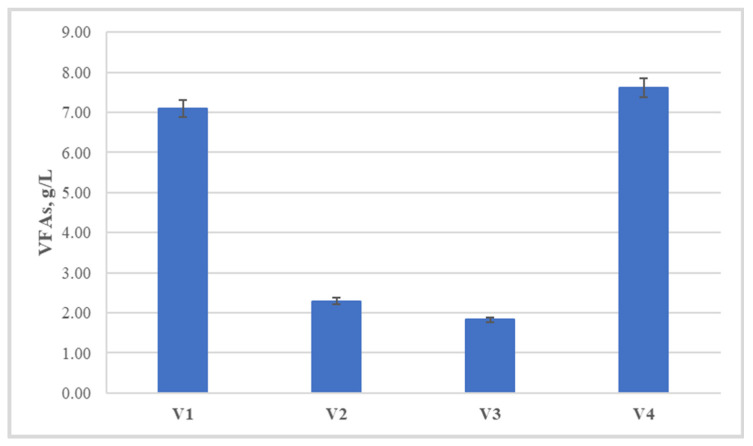
Total volatile fatty acids concentration at the end of the biomethane production process.

Due to the nature of the experiment being conducted, the possible reasons for the lower yields in two of the variants (V1 and V4) were analyzed after termination of the entire experiment in all variants. Quantitative and qualitative analysis of the volatile fatty acids present in the medium (Figure 6) unequivocally showed that one of the reasons for this is the high concentrations of these metabolites. Their total concentration for V1 and V4 reaches 7.1 and 7.6 g/L, respectively, in contrast to the more successful, in terms of methane yield, V2 and V3, with 2.3 and 1.8 g/L, respectively.

**Figure 7:**
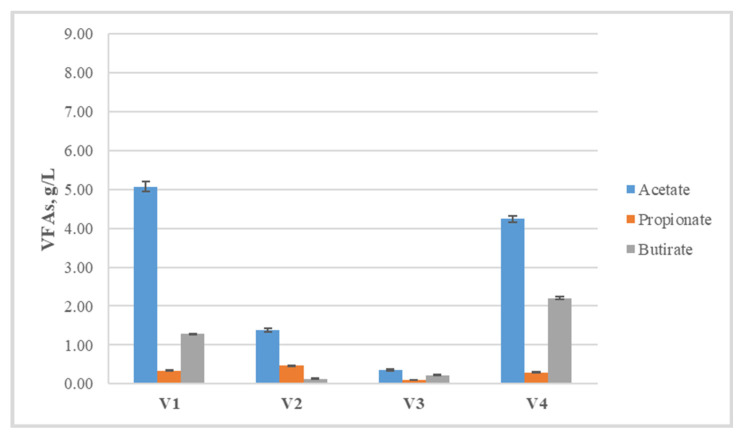
Concentrations of fatty acids with the highest content at the end of the methane production process.

The main precursors of methane production, acetate and propionate [35], present with high concentrations at the end of the process (Figure 7), which is a possible indication of an inhibited methanogenic process.

On a larger scale, a possible solution to overcome this problem would be monitoring and timely correction of the pH in the liquid fraction. Compared to the starting quantities, the acetate concentration shows a slight decrease in V4, while in V1, this concentration rises, which is probably a sign of an active hydrolysis process. Conversely, in variants V2 and V3, acetate concentrations are decreased, although not drastically. The ratio of propionate/acetate (Table 4) is far from the limit value (1.4), which can be used as a sign of impending termination of the process [36]. The values of longer chain fatty acids as well as isoforms are presented in Table 4.

**Figure 8:**
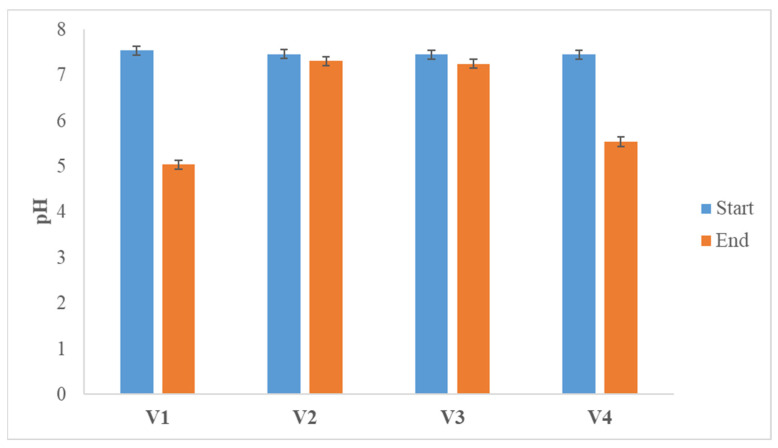
pH values at the start of methane production and at the end of the process.

Of these acids, in biomethane production, only caproate increases its concentration slightly. This increase in the concentration of acid metabolites, unlike the process of obtaining hydrogen, is reflected in the pH values and in the variants V1 and V4, which appear unsuitable for the methanogenesis phase (Figure 8).

According to the obtained results, the addition of waste cooking oil shows a positive effect on hydrogen production. However, it did not always show a negative relation to methane production. The impact is negative in the variant in which the lignocellulosic waste had undergone pretreatment. In this case, the accumulation of volatile fatty acids in concentrations that inhibit the activity of methanogens was registered.

Our research addresses a two-stage anaerobic digestion system where two energy carriers, hydrogen and methane, are obtained, which is most advantageous. Unlike with other fuels, methane and hydrogen combustion do not release any NOx (nitrous oxide) or SOx (sulphur dioxide), which are the major contributors to air pollution [37]. Hydrogen is recognized as a clean-energy fuel or fuel of the future since it does not release CO_2_ in the atmosphere during combustion.

A strong relation between process productivity and strictness has been established, maintaining the temperature and pH. A deviation of 10 °C in a mesophilic process leads to a reduction in the activity of microorganisms and their reproduction rate by 50%. Reducing the temperature by 20 °C leads to a complete failure of the process. Increase of the temperature above the optimum also leads to suppression of the process [38]. For an optimal range of pH values, 6.8–7.2 is indicated, but a decrease to 6.5 or increasing this value to 8.0 is permissible and does not lead to the suspension of the process [39].

Previously conducted processes at 35–37 °C showed that no biogas was produced with a substrate of untreated wheat straw, while at increased temperature, a biodegradation process was registered, accompanied by biogas release. These observations refer to processes with low dry matter content (liquid fermentation), while in this study, the interest is in realizing solid-phase processes at elevated temperature. Conducting the process of anaerobic digestion at 55 °C led to faster and higher hydrolysis rates and an increased degree of biodegradation, which was achieved in a stable system that enabled higher organic loading [40]. The choice of operating temperature as 55 °C was based on these effects, reported in our previous studies and those of other authors [41]. High-temperature conditions can also favor pathogen elimination, and the digestate obtained in such a process would more easily meet the sanitary/epidemiological requirements for its application as a biofertilizer [42]. The advantage of suppression of pathogenic microflora is also important when the residual unprocessed biomass is subsequently applied in plant breeding.

In a mesophilic process, pretreatment has a huge impact—the process simply does not start if the lignocellulosic material has not been pretreated. In this study, one of the objectives was to trace the possibility of applying untreated lignocellulosic feedstock (one of the assumptions is that pretreatment is an additional expenditure of raw materials and energy and is more difficult to implement under conditions of increased dry matter content). In the event we manage to achieve a good yield without pretreatment, by adding one or two additional and more easily degradable raw materials, then such a process would be interesting from the point of view practical application. 

**Figure 9:**
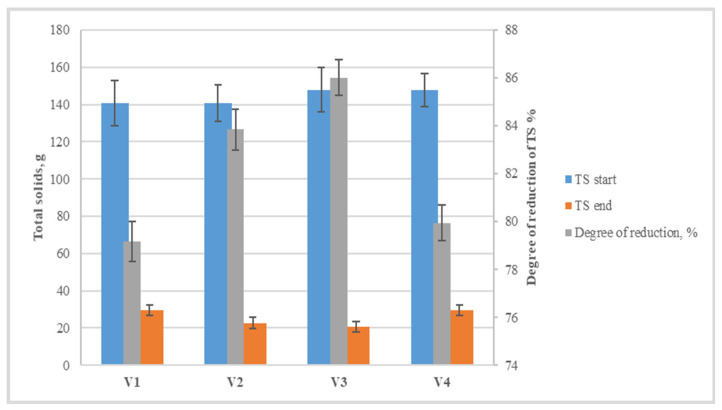
Degree of reduction of dry matter after two-step anaerobic digestion process for the production of hydrogen and methane.

At the end of the process, a reduction in total solids is registered in all variants. It is most significant in variants V2 and V3, reaching 84–86%. Despite the significantly lower methane yields, mainly in V1 and V4, the dry matter in these variants was also reduced by approximately 80% (Figure 9). These results can be explained by the total solids and volatile solids at the end of the process only being measured in the solid fraction—after the liquid fraction had been drained. It is possible that in variants with low yields of gaseous products, some of the organic matter is transformed from solid to liquid-soluble products, and these were carried away with the liquid fraction.

For the purpose of estimating the most appropriate variant, in our investigations, the energy yield was calculated in view of further scaling up the processes. Although the possibilities of obtaining energy in such small systems is not very effective because of the energy losses, the calculations presented in Table 5 serve as a final comparison to clarify which is the best option to be used in the future when scaling up to larger volume systems. The lower energy yields in options V1 and V4 can be attributed to the lack of pre-treatment of the wheat straw in V1 and the inhibitory effect of secondary metabolites as a result of the cooking oils’ addition in V4. Although in the first phase of the process, the production of hydrogen, variant V2 has a lower yield than V4, the final calculations showed that it is in V2 where the highest yield of energy is realized from the two energy carriers. This is mainly due to the significantly higher methane yield in this variant compared to all other variants. The difference between the energy yield of this variant and the variant that followed (V3) was four-fold. As for the application of waste cooking oil, it is clear from Table 5, with the calculated energy equivalents, that the addition of waste cooking oil to a process with untreated lignocellulose leads to an increase in yield of about 19 times (V1 vs. V3), although this result is about 4 times lower compared to the variant without the addition of waste cooking oil but with pretreatment (V2 vs. V3).

Despite the low contribution of hydrogen to the total energy of the process, the application of two-step processes can be practically useful because the accelerated hydrolysis can lead to a shortening of the total biodegradation time per unit of feedstock, and the gas mixture of methane and hydrogen (biohytane) has higher caloric value.

Separation of the stages in anaerobic digestion represents a promising strategy for the flexibilization of the fermentative part of biogas production. It allows an easier combination of material and energy use of residual biomass sources, coupled with effluent recirculation [43]. Process phase separation can ensure optimal conditions for every process phase, which can be controlled separately in each stage.

## 4. Conclusions

The demand for renewable fuels is growing, along with concern about climate change, nature preservation, energy dependence, and the depletion of fossil fuels. The suggested dry anaerobic co-digestion process with two stages reveals the possibilities for the development of technologies that can be successfully incorporated in the circular economy concept. Biogas is one of the versatile renewable fuels used for power production. In this study, along with the production of hydrogen and methane and the separation of the hydrolysis and methanogenesis stages, the utilization of agriculture- and kitchen-generated wastes was realized in the context of waste-to-energy sustainable production methods. The use of waste cooking oil as a co-substrate showed that it acted as a positive modulator of anaerobic digestion for hydrogen production, and at the applied concentration of 5%, it did not inhibit the hydrolysis phase. Hydrogen production as a first phase of the process was realized without pH correction needed. Acetate concentrations of 1.4 g/L and total volatile fatty acids concentration of 2.2 g/L showed no inhibitory effect on the methanogenesis process. The overall efficiency of the two-stage anaerobic digestion of the mixture, which contained mainly lignocellulose waste, is positively dependent on the thermochemical pretreatment with the alkali reagent (Ca(OH)_2_), but elevated temperature (55 °C) and cooking oil addition revealed the opportunity to omit the pretreatment step. Nevertheless, the overall energy production was lower due to the methane production step. However, the addition of waste cooking oils to the process in which lignocellulose is not pretreated (V3) led to an increase in the methane production and energy yield compared to V1. A two-stage anaerobic digestion of the mixture of waste wheat straw, horse manure, and cooking oil was accomplished, and the opportunity to omit the pretreatment step was proven.

## Figures and Tables

**Table 1:** Variants for testing the effect of pretreatment and addition of waste cooking oil to a mixture of wheat straw and horse manure on the potential to produce hydrogen and methane.

Variant	Pretreatment	Added Waste Cooking Oil, %
V1	No	No
V2	4% (wt.) Ca(OH)_2_, 55 °C/24 h	No
V3	No	5
V4	4% (wt.) Ca(OH)_2_, 55 °C/24 h	5

**Table 2:** Characteristics of the raw materials used.

Indicator	Type of Raw Material Used	
	Wheat Straw	Horse Manure
Moisture content, %	7.64 ± 0.2	63.29 ± 1.6
Density, kg/m^3^	152.0 ± 5.7	518.9 ± 28.1
Total solids, %	92.36 ± 1.8	36.71 ± 3.8
Volatile solids, % TS	89.58 ± 1.3	47.87 ± 1.7
Cellulose, % TS	37.6 ± 2.4	28.7 ± 2.2

**Table 3:** Concentrations of the minor fatty acids in the anaerobic digestion process for hydrogen production.

Volatile Fatty Acids, g/L					
Variant	i-Butyrate	i-Valerate	Valerate	Caproate	Propionate/Acetate
V1	0	0	0	0.15 ± 0.01	0.13
V2	0.07 ± 0.01	0.07 ± 0.01	0.08 ± 0.01	0.18 ± 0.01	0.06
V3	0	0	0	0.09 ± 0.01	0.09
V4	0.07 ± 0.01	0.07 ± 0.01	0.09 ± 0.01	0.24 ± 0.01	0.05

**Table 4:** Concentrations of the minor fatty acids in the anaerobic digestion process for methane production.

Volatile Fatty Acids, g/L					
Variant	i-Butyrate	i-Valerate	Valerate	Caproate	Propionate/Acetate
V1	0	0	0.09 ± 0.01	0.31 ± 0.02	0.07
V2	0.17 ± 0.01	0.15 ± 0.01	0	0	0.34
V3	0.34 ± 0.02	0.18 ± 0.01	0.11 ± 0.01	0.51 ± 0.02	0.29
V4	0.09 ± 0.01	0.08 ± 0.01	0.15 ± 0.01	0.57 ± 0.02	0.07

**Table 5:** Comparison of the total potential energy yield for the studied variants.

	Energy Carrier	Total Yield, N cm^3^	Y, cm^3^·g^−1^	Lower Heating Value, KWh·N cm^3^	Total Energy for Corresponding Energy Carrier, kWh·t^−1^	Total Energy for the System, kWh·t^−1^
V1	Hydrogen	22.5	0.08	2.99	0.17	0.37
	Methane	7.51	0.03	9.94	0.20	
V2	Hydrogen	73.34	0.29	2.99	0.56	30.85
	Methane	1072.73	4.20	9.94	30.29	
V3	Hydrogen	25.87	0.10	2.99	0.19	7.11
	Methane	257.16	0.98	9.94	6.92	
V4	Hydrogen	96.42	0.37	2.99	0.71	0.83
	Methane	4.28	0.02	9.94	0.12	

## Data Availability

Data are contained within the article.

## References

[1] Rawoof S.A.A., Kumar P.S., Vo D.V.N., Subramanian S. (2021). Sequential production of hydrogen and methane by anaerobic digestion of organic wastes: A review. Environ. Chem. Lett..

[2] Akturk A.S., Demirer G.N. (2020). Improved Food Waste Stabilization and Valorization by Anaerobic Digestion Through Supplementation of Conductive Materials and Trace Elements. Sustainability.

[3] Sevillano C.A., Pesantes A.A., Peña Carpio E., Martínez E.J., Gómez X. (2021). Anaerobic Digestion for Producing Renewable Energy—The Evolution of This Technology in a New Uncertain Scenario. Entropy.

[4] Hou H., Lu W., Liu B., Hassanein Z., Mahmood H., Khalid S. (2023). Exploring the Role of Fossil Fuels and Renewable Energy in Determining Environmental Sustainability: Evidence from OECD Countries. Sustainability.

[5] Łukajtis R., Hołowacz I., Kucharska K., Glinka M., Rybarczyk P., Przyjazny A., Kamiński M. (2018). Hydrogen production from biomass using dark fermentation. Renew. Sustain. Energy Rev..

[6] Yeshanew M.M., Paillet F., Barrau C., Frunzo L., Lens P.N.L., Esposito G., Escudie R., Trably E. (2018). Co-production of Hydrogen and Methane From the Organic Fraction of Municipal Solid Waste in a Pilot Scale Dark Fermenter and Methanogenic Biofilm Reactor. Front. Environ. Sci..

[7] Barcelos M.C.S., Lupki F.B., Campolina G.A., Nelson D.L., Molina G. (2018). The colors of biotechnology: General overview and developments of white, green and blue areas. FEMS Microbiol. Lett..

[8] Mahapatra S., Kumar D., Singh B., Sachan P.K. (2021). Biofuels and their sources of production: A review on cleaner sustainable alternative against conventional fuel, in the framework of the food and energy nexus. Energy Nexus.

[9] Gao Z., Alshehri K., Li Y., Qian H., Sapsford D., Cleall P., Harbottle M. (2022). Advances in biological techniques for sustainable lignocellulosic waste utilization in biogas production. Renew. Sustain. Energy Rev..

[10] Simeonov I.S., Denchev D.D., Kabaivanova L.V., Kroumova E.T.Z., Chorukova E.Y., Hubenov V.N., Mihailova S.N. (2017). Different types of pre-treatment of lignocellulosic wastes for methane production. Bulg. Chem. Commun..

[11] Yue Z.B., Li W.W., Yu H.Q. (2013). Application of rumen microorganisms for anaerobic bioconversion of lignocellulosic biomass. Bioresour. Technol..

[12] Panadare D.C., Rathod V.K. (2015). Applications of Waste Cooking Oil Other than Biodiesel: A Review. Iran. J. Chem. Eng..

[13] Hanum F., Yuan L.C., Kamahara H., Aziz H.A., Atsuta Y., Yamada T., Daimon H. (2019). Treatment of Sewage Sludge Using Anaerobic Digestion in Malaysia: Current State and Challenges. Front. Energy Res..

[14] Rouches E., Escudié R., Latrille E., Carrère H. (2019). Solid-state anaerobic digestion of wheat straw: Impact of S/I ratio and pilot-scale fungal pretreatment. Waste Manag..

[15] Kabaivanova L., Hubenov V., Dimitrova L., Simeonov I., Wang H., Petrova P. (2022). Archaeal and Bacterial Content in a Two-Stage Anaerobic System for Efficient Energy Production from Agricultural Wastes. Molecules.

[16] Amodeo C., Hattou S., Buffiere P., Benbelkacem H. (2021). Temperature phased anaerobic digestion (TPAD) of organic fraction of municipal solid waste (OFMSW) and digested sludge (DS): Effect of different hydrolysis conditions. Waste Manag..

[17] Denchev D., Hubenov V., Simeonov I., Kabaivanova L. Biohydrogen production from lignocellulosic waste with anaerobic bacteria. Proceedings of the 4th International Conference on Water, Energy and Environment (ICWEE).

[18] Updegraff D. (1969). Semimicro determination of cellulose inbiological materials. Anal. Biochem..

[19] Sluiter A., Ruiz R., Scarlata C., Sluiter J., Templeton D. (2008). Determination of Extractives in Biomass: Laboratory Analytical Procedure (LAP); Issue Date 7/17/2005. https://www.nrel.gov/docs/gen/fy08/42619.pdf.

[20] Van Wychen S., Laurens L.M.L. (2016). Determination of Total Solids and Ash in Algal Biomass: Laboratory Analytical Procedure (LAP).

[21] Dell’Omo P.P., Spena V.A. (2020). Mechanical pretreatment of lignocellulosic biomass to improve biogas production: Comparison of results for giant reed and wheat straw. Energy.

[22] Mañunga T., Barrios-Pérez J.D., Zaiat M., Rodríguez-Victoria J.A. (2022). Evaluation of pretreatment methods and initial pH on mixed inoculum for fermentative hydrogen production from cassava wastewater. Biofuels.

[23] Kothari R., Pandey A.K., Kumar S., Tyagi V.V., Tyagi S.K. (2014). Different aspects of dry anaerobic digestion for bio-energy: An overview. Renew. Sustain. Energy Rev..

[24] Anwar Z., Gulfraz M., Irshad M. (2014). Agro-industrial lignocellulosic biomass a key to unlock the future bio-energy: A brief review. J. Radiat. Res. Appl. Sci..

[25] Naji A., Rechdaoui S.G., Jabagi E., Lacroix C., Azimi S., Rocher V. (2023). Horse Manure and Lignocellulosic Biomass Characterization as Methane Production Substrates. Fermentation.

[26] Niju S., Swathika M., Balajii M., Yousuf A., Pirozzi D., Sannino F. (2020). Chapter 10—Pretreatment of lignocellulosic sugarcane leaves and tops for bioethanol production. Lignocellulosic Biomass to Liquid Biofuels.

[27] Awasthi M.K., Sar T., Gowd S.C., Rajendran K., Kumar V., Sarsaiya S., Li Y., Sindhu R., Binod P., Zhang Z. (2023). A comprehensive review on thermochemical, and biochemical conversion methods of lignocellulosic biomass into valuable end product. Fuel.

[28] Selvi P.K., Sharma M., Kamyotra J.S. (2013). Spent oil management and its recycling potential in India inventory and issues. Procedia Environ. Sci..

[29] Ortner M.E., Müller W., Schneider I., Bockreis A. (2016). Environmental assessment of three different utilization paths of waste cooking oil from households. Resour. Conserv. Recycl..

[30] Lopes M., Miranda S.M., Belo I. (2020). Microbial valorization of waste cooking oils for valuable compounds production—A review. Crit. Rev. Environ. Sci. Technol..

[31] Sillero L., Solera R., Perez M. (2023). Effect of temperature on biohydrogen and biomethane production using a biochemical potential test with different mixtures of sewage sludge, vinasse and poultry manure. J. Clean. Prod..

[32] Zerback T., Schumacher B., Weinrich S., Hülsemann B., Nelles M. (2022). Hydrothermal Pretreatment of Wheat Straw—Evaluating the Effect of Substrate Disintegration on the Digestibility in Anaerobic Digestion. Processes.

[33] Qu X., Zeng H., Gao Y., Mo T., Li Y. (2022). Bio-hydrogen production by dark anaerobic fermentation of organic wastewater. Front. Chem..

[34] Dubrovskis V., Plume I., Straume I. (2018). Anaerobic co-fermentation of molasses and oil with straw pellets. Agron. Res..

[35] Zhou M., Yan B., Wong J.W.C., Zhang Y. (2018). Enhanced volatile fatty acids production from anaerobic fermentation of food waste: A mini-review focusing on acidogenic metabolic pathways. Bioresour. Technol..

[36] Buyukkamaci N., Filibeli A. (2004). Volatile fatty acid formation in an anaerobic hybrid reactor. Process Biochem..

[37] Gaffney J.S., Marley N.A. (2009). The impacts of combustion emissions on air quality and climate—From coal to biofuels and beyond. Atmos. Environ..

[38] Cioabla A.E., Ionel I., Dumitrel G.A., Popescu F. (2012). Comparative study on factors affecting anaerobic digestion of agricultural vegetal residues. Biotechnol. Biofuels.

[39] Hubenov V., Miteva-Staleva J., Eneva R., Boteva N., Kabaivanova L. (2021). Two-stage anaerobic digestion of wheat straw using immobilized microbial consortia. Ecol. Eng. Environ. Prot..

[40] Kabaivanova L., Petrova P., Hubenov V., Simeonov I. (2022). Biogas Production Potential of Thermophilic Anaerobic Biodegradation of Organic Waste by a Microbial Consortium Identified with Metagenomics. Life.

[41] Singh R., Hans M., Kumar S., Yadav Y.K. (2023). Thermophilic Anaerobic Digestion: An Advancement towards Enhanced Biogas Production from Lignocellulosic Biomass. Sustainability.

[42] Börjesson P., Mattiasson B. (2008). Biogas as a resource-efficient vehicle fuel. Trends Biotechnol..

[43] Van D.P., Fujiwara T., Tho B.L., Toan P.P.S., Minh G.H. (2020). A Review of Anaerobic Digestion Systems for Biodegradable Waste: Configurations, Operating Parameters, and Current Trends. Environ. Eng. Res..

